# Comparative efficacy of advanced therapeutic modalities for diabetic foot ulcers: a systematic review and meta-analysis including NPWT, stem cells, ESWT, and bioengineered treatments

**DOI:** 10.3389/fbioe.2026.1792670

**Published:** 2026-04-29

**Authors:** Xiaofang Guan, Guangyong Zhu, Haowei Jiang, Bo Wang, Jiaxin Cheng

**Affiliations:** 1 Hand and Foot Surgery, The First People’s Hospital of Xiaoshan District, Xiaoshan, China; 2 Department of Orthopedics, Hangzhou Hospital of Zhejiang Medical Health Group, Hangzhou, China

**Keywords:** DFU, diabetic foot ulcers, exosomes, extracorporeal shockwave therapy, mesenchymal stem cells, MSc, negative pressure wound therapy, Npwt

## Abstract

**Background:**

Diabetic foot ulcers (DFUs) are highly debilitating and often necessitate cutting-edge treatments apart from the usual care. This systematic review and meta-analysis have compared the efficacy of Negative Pressure Wound Therapy (NPWT)/VAC, cell-based therapies (MSC-based therapies), artificial skin, extracorporeal shockwave therapy (ESWT), and platelet-rich plasma (PRP) interventions in promoting healing of difficult wounds, as well as healing by other means.

**Methods:**

The extensive search identified 49 randomized controlled trials. For data analysis, a random-effects model with inverse-variance weighting was used to obtain standardized mean differences (SMDs) with 95% confidence intervals (CIs). The presence of publication bias was determined using funnel plots and Egger’s test, while the quality of the studies was assessed by means of Jadad scores and risk of bias tools. GRADE was employed to assess the certainty of the evidence.

**Results:**

Sub-group analysis revealed that NPWT significantly accelerated wound closure compared to standard care SMD of −0.39 (95% CI: 0.57 to −0.21; I^2^ = 49%), indicating faster healing times, used days to measure time showed an SMD of −0.94 (95% CI: 1.07 to −0.80; I^2^ = 0%) which showed that patients healed at a faster rate. The comparison of different devices and techniques showed an SMD of 0.98 (95% CI: 0.75–1.21; I^2^ = 0%), indicating that additional treatments resulted in a greater reduction in wound area. The results were statistically significant because all p-values were lower than 0.05. MSC-based therapies showed similar overall healing (SMD = 0.46, 95% CI: 0.24–0.69) and faster closure (SMD = −1.04, 95% CI: 1.28 to −0.8). Additionally, trials of bioengineered skin substitutes, ESWT, and PRP all showed statistically significant improvements in time to wound closure and overall healing. Relative to the control group, adverse events were fewer in the NPWT and PRP groups (SMD = −0.24, 95% CI: 0.42 to −0.05).

**Conclusion:**

The NPWT and MSC-based therapy, in particular, represent major improvements in healing outcomes for diabetic foot ulcers (DFUs) and in the speed of closure compared with standard treatment.

## 1 Introduction

Diabetic foot ulcers (DFUs) are probably the worst and most serious complications of diabetes mellitus that occur in 15%–25% diabetic patients at least once in their lives. Besides, they have a huge economic impact, being a major cause of hospitalization and the costs associated with the treatment of infected wounds and amputations. Hyperglycemia, characteristic of diabetes nowadays, is a main factor that causes the development of neuropathy, vascular diseases, and the breakdown of the immune system, thus creating a very complicated and unstimulating mechanism for healing the wound.

The occurrence of DFUs is often a result of ischemia, infection, and stress on the affected area, which together prolong healing and increase the risk of ulcer recurrence. Hence, proper management of DFUs always involves a multidisciplinary team that simultaneously addresses the underlying metabolic problems and utilizes the latest medical technologies to promote healing and prevent complications. The conventional treatment of diabetic foot ulcers (DFUs) has primarily focused on wound management, emphasizing debridement, offloading, infection control, and the use of moist dressings (Lev-Tov et al., 2013; Viswana et al., 2020; Gupta et al., 2021; Smith et al., 2020; Elsaid et al., 2020; Xu et al., 2018).

Negative Pressure Wound Therapy (NPWT), or vacuum-assisted closure (VAC), has gained recognition as the most effective technique among these due to its ability to apply controlled mechanical stress, enhance blood flow, reduce swelling, and remove fluid. Extensive research has found that NPWT not only increases the rate of complete wound healing but also decreases the time to closure, and, in general, it is safe. However, device-related incidents and costs still need to be taken into account. Stem cell therapies, such as mesenchymal stem cells (MSCs) and exosome-derived treatments, constitute a new strategy that not only effectively counter the cellular and molecular deficits but also directly target the fundamental causes of diabetes-related impaired wound healing (Park et al., 2018; Bhansali et al., 2009; Khandelwal et al., 2013; Ahmed et al., 2017).

Extracorporeal shockwave therapy (ESWT) and other physical treatment modalities have been evaluated for the treatment of diabetic foot ulcers (DFUs) (Wu et al., 2024). The ESWT procedure uses focused sound waves to create new blood vessels, accelerate cell division, and alter immune responses. The results from the clinic have shown that the use of ESWT can reduce healing time and, at the same time, increase the rate of wound specifications achieving complete closure, particularly when used alongside regular wound care (Zhang et al., 2018). Another example of an innovative method that has demonstrated effectiveness in treating DFUs is platelet-rich plasma (PRP) therapy, which involves the concentrated application of one’s own platelets and growth factors (Yang et al., 2025; Syafira et al., 2024). The present systematic review and meta-analysis aim to synthesize existing evidence on NPWT, stem cell- and MSC-based therapies, bioengineered skin substitutes, ESWT, and platelet-based interventions for DFUs.

## 2 Methodology

The systematic review and meta-analysis were performed according to the PRISMA guidelines, which help to maintain transparency and reproducibility. The aim was to compare the effectiveness of various advanced therapeutic modalities, including Negative Pressure Wound Therapy (NPWT), stem cell- and MSC-based interventions, bioengineered skin substitutes, extracorporeal shockwave therapy (ESWT), and platelet-rich plasma (PRP)-based treatments, in the management of diabetic foot ulcers (DFUs).

### 2.1 Search strategy and study selection

The literature search was conducted in a very thorough manner, spanning electronic databases including PubMed, Embase, the Cochrane Library, Web of Science, and Scopus, and covering the period from the start of each database to the end of December 2025. The search terms went through various combinations such as “diabetic foot wound,’ ‘DFU,’ or ‘diabetic foot lesion,” “NPWT’ or ‘vacuum-assisted closure,” “vacuum-assisted closure,” “stem cells,” “mesenchymal stem cells,” “exosomes,” “bioengineered skin,” “collagen,” “growth factor,” “extracorporeal shockwave therapy,” “ESWT,” “platelet-rich plasma,” “PRP,” and “randomized controlled trial.” Furthermore, the reference lists of pertinent articles and previous reviews were inspected for additional studies.

The studies ultimately selected were RCTs and clinical studies involving adults with DFUs and assessing the aforementioned advanced interventions. The studies needed to report at least one of the primary outcomes, which included complete wound healing (proportion healed), time to wound closure, wound area reduction, or adverse events (including infections or amputations). Publications in languages other than English, case reports, reviews, and studies without extractable data were not considered. Data from gray literature were not included in the systematic review.

### 2.2 Data extraction

A pair of independent reviewers screened titles, abstracts, and full texts to identify eligible studies. Any differences of opinion were resolved either by agreement or through the intervention of a third reviewer. The extracted data consisted of study characteristics (author, year, country), sample size, details of the intervention and comparator, ulcer type and grade, duration of follow-up, and outcome measures. Continuous data (e.g., time to closure, wound area reduction) were reported as mean and standard deviation (SD). For binary outcomes, proportions were converted to means and SDs to enable pooling in the meta-analysis.

### 2.3 Quality assessment

The quality of included studies was evaluated on a methodological basis by means of the Jadad scale, which measured randomization, blinding, and reporting of withdrawals/dropouts. The risk of bias was again assessed independently using the Cochrane Collaboration’s Risk of Bias 2.0 tool, which assesses five types of potential bias: the randomization process, deviations from intended interventions, missing outcome data, measurement of outcomes, and selection of reported results. The certainty of evidence for each outcome was graded according to the GRADE (Grading of Recommendations, Assessment, Development, and Evaluation) framework, taking into account risk of bias, inconsistency, indirectness, imprecision, and publication bias.

### 2.4 Data synthesis and statistical analysis

Meta-analyses were carried out using a random-effects model and the inverse-variance method to account for between-study heterogeneity. SMDs (standardized mean differences) were calculated for continuous variables, and proportions were calculated for dichotomous variables. I_1_ was used to measure heterogeneity, with I^2^ values > 50% indicating high heterogeneity, a common practice. The presence of publication bias was assessed both visually and statistically, the former using funnel plots and the latter using Egger’s regression test. In addition, for each intervention type and outcome measure, subgroup analyses were conducted to identify differences in efficacy and safety.

Statistical testing was performed using Review Manager (RevMan) version 5.4 and STATA version 17. The results were presented using pooled effect sizes and their corresponding 95% confidence intervals (CIs), with statistical significance set at p < 0.05. Furthermore, sensitivity analyses were conducted in which studies with a high risk of bias or small sample sizes were excluded, allowing us to evaluate the robustness of the findings.

The methodology employed ensures a comprehensive, unambiguous, and open evaluation of the relative effectiveness of new therapeutic modalities for diabetic foot ulcers (DFUs), thereby providing clinicians and researchers with high-quality evidence specifically designed for clinical decision-making.

## 3 Results

### 3.1 Study selection

**FIGURE 1:**
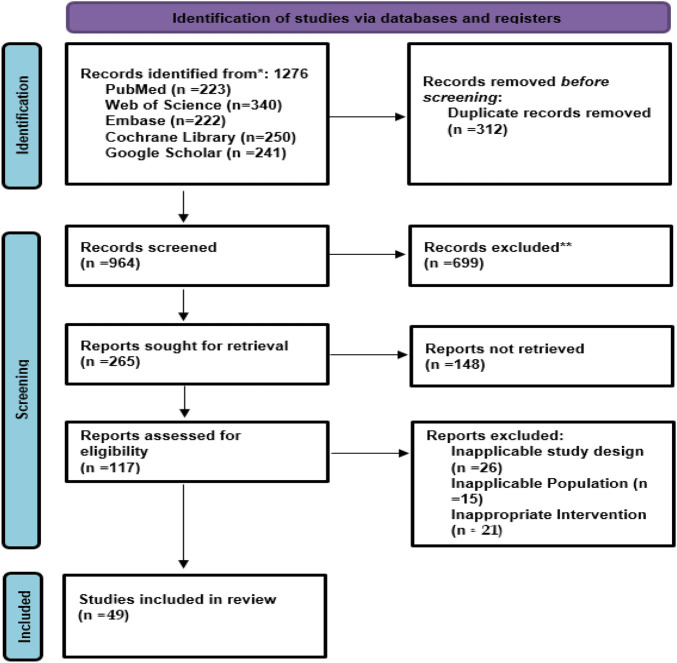
PRISMA flow chart of study selection

The initial literature search yielded a total of 1,276 articles from databases including PubMed, Embase, the Cochrane Library, Web of Science, and Scopus. After that, the screening of titles and abstracts was conducted on the 964 records remaining after the removal of 312 duplicates. Out of the 964, 847 studies were eliminated because they were either irrelevant or did not involve comparative designs or failed to meet the inclusion criteria (e.g., case reports, reviews, studies of non-humans). A total of 117 articles were selected to be read in their entirety for the determination of their eligibility. Within this phase, 65 studies were excluded for various reasons: lack of outcome data (n = 28), non-RCT or non-comparative design (n = 19), use of interventions that were not among the defined advanced modalities (NPWT, MSC-based therapies, ESWT, bioengineered skin substitutes, PRP) (n = 12), and overlapping cohorts (n = 6). In the end, 49 studies were included in the systematic review and meta-analysis (Figure 1).

### 3.2 Baseline characteristics

The systematic review and meta-analysis included 49 studies involving more than 6,500 adult patients with diabetic foot ulcers (DFUs) treated in countries such as the USA, China, Germany, India, and Egypt. The major study methods were randomized controlled trials (RCTs) and multicenter, prospective comparative studies. The sample sizes ranged from 30 to 507 people. The treatments applied were negative pressure wound therapy (NPWT), single-use and low-cost NPWT, MSC-based therapies, extracellular shockwave therapy (ESWT), autologous platelet-rich plasma (PRP), and bioengineered skin substitutes (dHACM, EpiCord, Grafix®), topical growth factors (EGF, PDGF-BB), and advanced dressings (silver nanoparticles, copper-based). The duration of treatment ranged from 2 to 24 weeks, depending on the case. In the majority of cases, the antibiotics were neuropathic or neuro-ischemic with sufficient perfusion; ischemic or post-amputation wounds were selectively included. The studies consistently showed that advanced modalities not only achieved faster wound closure but also demonstrated better granulation, reduced infection, and, in some instances, lower resource utilization compared with standard care, thereby highlighting the heterogeneity and therapeutic potential of modern DFU interventions (Table 1).

**TABLE 1:** Baseline characteristics of the included studies.

Author(s) Year	Country	Study type & population	Sample size (total)	Control size	Intervention	Treatment duration	Vascular/Anatomic factors	Outcomes
Blume et al. (2008)	Multinational (USA-led)	Multicenter RCT; adults with DFUs	342	169 (AMWT)	NPWT (VAC)	≤16 weeks/until closure	Neuropathic/neuro-ischemic; adequate perfusion	Higher closure rate, faster healing
Gu et al. (2025)	China	Comparative clinical study; DFUs	198	99 (AMWT)	NPWT	4–8 weeks	Mainly neuropathic; no critical ischemia	Improved healing, less infection
Malekpour et al. (2021)	Iran	RCT; infected DFUs	60	30 (conventional)	NPWT	2–4 weeks	Wagner II–III; ischemia excluded	Faster infection control, granulation
Zelen et al. (2016)	Multinational	Prospective multicenter RCT; chronic DFUs	126	63 (advanced care)	Advanced therapies incl. NPWT	≤12 weeks	Chronic neuropathic; adequate flow	Higher healing, cost-effectiveness
Kirsner et al. (2021)	United States	Prospective RCT; DFUs	164	82 (traditional NPWT)	Single-use NPWT	≤12 weeks	Adequate perfusion	Non-inferior healing, fewer resources
Seidel et al. (2022)	Germany	RCT (DiaFu); DFUs	368	184 (SMWC)	NPWT	16 weeks	Mixed neuropathic/ischemic	Similar healing; higher resource use
Davis et al. (2020)	United States of America	RCT; complex foot infections	120	60 (standard NPWT)	NPWT + saline irrigation	2–6 weeks	Deep infected wounds	Better infection control
Kirsner et al. (2019)	United States	Prospective RCT; chronic ulcers	165	83 (traditional NPWT)	Single-use NPWT	≤12 weeks	DFUs/venous; adequate flow	Non-inferior healing
Armstrong et al. (2005)	Multinational	Multicenter RCT; post-amputation DFUs	162	85 (standard care)	NPWT	16 weeks	Post-partial amputation	Faster closure
Wu et al. (2023)	China	RCT; DFUs	102	51 (moist dressing)	NPWT	4 weeks	Neuropathic; no ischemia	Better wound bed prep
Shukr and Ahmed (2015)	Pakistan	RCT; DFUs	140	70 (AMWT)	NPWT (VAC)	8–12 weeks	Wagner I–III; ischemia excluded	Faster healing
Sepúlveda et al. (2009)	Spain	RCT; diabetic foot amputation wounds	70	35 (standard dressing)	NPWT	4–8 weeks	Post-amputation wounds; perfusion assessed	Faster healing and fewer complications
James et al. (2019)	India	RCT; DFUs	60	30 (conventional)	VAC therapy	4 weeks	Neuropathic DFUs; ischemia excluded	Improved healing rate and granulation
Seidel et al. (2020)	Germany	Pragmatic RCT (DiaFu); DFUs	345	172 (SMWC)	NPWT	≤16 weeks	Real-world DFUs; mixed etiology	No superiority; higher costs
Melamed et al. (2025)	Israel	RCT; diabetic wounds	156	78 (copper dressings)	NPWT	12 weeks	Chronic DFUs; adequate perfusion	Copper dressings are non-inferior to NPWT
Armst et al. (2012)	Multinational	Multicenter RCT; DFUs	130	65 (alt. NPWT device)	Mechanical vs. electrical NPWT	≤12 weeks	Chronic DFUs; perfusion adequate	Comparable efficacy across devices
Dsouza et al. (2017)	India	RCT; DFUs	80	40 (conventional)	Low-cost VAC	4–6 weeks	Neuropathic DFUs	Improved healing at lower cost
Snyder et al. (2018)	Multinational	Two multicentre, double-blinded phase III RCTs; DFUs	336	168 (sham/control)	Focused shockwave therapy	12 weeks	Chronic DFUs; adequate perfusion	Higher complete closure vs. control
Elsaid et al. (2020)	Egypt	RCT; non-healing DFUs	80	40 (saline dressing)	Autologous PRP dressing	8 weeks	Chronic DFUs; no critical ischemia	Faster healing, greater ulcer size reduction
Zhong et al. (2024)	China	Prospective RCT; DFUs with osteomyelitis	98	49 (NPWT alone)	NPWT + antibiotic bone cement	6–8 weeks	Deep DFUs; bone involvement	Accelerated healing, better infection control
Essa et al. (2023)	Egypt	Prospective RCT; DFUs	90	45 (conventional dressing)	Silver nanoparticle dressing	6 weeks	Neuropathic DFUs	Improved healing rate
Kr (2018)	India	Comparative clinical study; DFUs	60	30 (conventional dressing)	NPWT	4–6 weeks	Wagner grade I–III DFUs	Faster granulation and closure
Taha et al. (2023)	Egypt	RCT; surgically treated DFU infections	84	42 (standard care)	NPWT	2–4 weeks	Post-debridement infected DFUs	Reduced complications, faster healing
Srivastava et al. (2022)	India	RCT; DFUs	60	30 (standard care)	NPWT	4 weeks	Neuropathic DFUs	Reduced wound temperature, better healing
Maranna et al. (2021)	India	RCT; grade I–II DFUs	100	50 (conventional dressing)	NPWT	4 weeks	Early-stage neuropathic DFUs	Faster healing and granulation
Vaidhya et al. (2015)	India	Prospective comparative study; DFUs	50	25 (conventional dressing)	Low-cost NPWT (indigenously designed)	4–6 weeks	Neuropathic DFUs; adequate perfusion	Comparable healing to standard NPWT at a lower cost
Adham et al. (2022)	Egypt	RCT; postoperative DFU wounds	80	40 (conventional dressing)	NPWT	2–4 weeks	Post-surgical DFU wounds; perfusion assessed	Faster wound healing and fewer complications
Al-Mallah et al. (2018)	Egypt	RCT; DFU wounds	60	30 (conventional dressing)	NPWT	4 weeks	Neuropathic DFUs; ischemia excluded	Greater wound size reduction and granulation
Sajid et al. (2015)	Pakistan	RCT; DFUs	140	70 (AMWT)	a NPWT (VAC)	8–12 weeks	Wagner grade I–III DFUs; ischemia excluded	Faster healing and improved ulcer closure
Kishta et al. (2025)	Egypt	RCT; chronic DFUs	90	45 (standard care)	Wharton’s jelly MSC-derived exosomes	12 weeks	Chronic DFUs; adequate vascular supply	Accelerated healing and improved tissue regeneration
Zollino (2017)	Italy	Phase II RCT; chronic leg ulcers*	30	15 (standard care)	Adipose-derived stem cell therapy	12–24 weeks	Recalcitrant chronic ulcers*	Improved healing rates vs. control
Zelen et al. (2015)	Multinational	Multicenter RCT; chronic DFUs	100	33 (SOC)	dHACM allograft vs. bioengineered skin substitute	12 weeks	Chronic neuropathic DFUs; adequate perfusion	Higher healing rates with dHACM
Narayan et al. (2025)	India	Multicenter RCT; DFUs	120	60 (collagen substitute)	dHACM allograft	12 weeks	Neuropathic DFUs; ischemia excluded	Comparable or superior healing efficacy
Tettelbach et al. (2019)	Multinational	Multicenter RCT; DFUs	155	77 (standard care)	Dehydrated human umbilical cord allograft (EpiCord)	12 weeks	Chronic DFUs; adequate arterial supply	Higher complete closure rates
Veves et al. (2002)	Multinational (USA-led)	RCT; chronic DFUs	276	138 (standard care)	Promogran® (collagen/ORC dressing)	12 weeks	Neuropathic DFUs; adequate perfusion	Similar overall healing; benefit in deeper ulcers
Gunasekaran (2026)	India and United States	Dual RCTs; DFUs	55	27 (comparator)	Type I collagen skin substitute	12 weeks	Chronic DFUs; ischemia excluded	Comparable or superior healing vs. amniotic/chorion
Mulder et al. (2014)	United States	Prospective comparative study; DFUs	64	32 (standard care)	Advanced wound modalities (biologics/devices)	12 weeks	Chronic DFUs; perfusion assessed	Reduced time to healing
Lavery et al. (2014)	Multinational	Multicenter blinded RCT; chronic DFUs	97	50 (SOC)	Grafix® (cryopreserved placental membrane)	12 weeks	Chronic neuropathic DFUs; adequate perfusion	Higher complete closure rates; safe
Tan et al. (2023)	Indonesia	Phase II clinical trial; chronic wounds*	60	30 (standard care)	hUCMSC secretome gel (10%)	8–12 weeks	Diabetic and trophic ulcers*	Improved healing and granulation
Omar et al. (2014)	Saudi Arabia	Single-blinded RCT; chronic DFUs	60	30 (standard care)	Shock wave therapy	6 weeks	Chronic neuropathic DFUs	Faster healing and ulcer size reduction
Vangaveti et al. (2023)	Australia	Prospective RCT; DFUs	90	45 (standard care)	Extracorporeal shockwave therapy	8 weeks	Chronic DFUs; adequate perfusion	Improved healing rate and pain reduction
Gupta et al. (2021)	India	RCT; DFUs	80	40 (standard care)	Intralesional platelet-rich plasma	6 weeks	Chronic DFUs	Faster healing and ulcer size reduction
Smith et al. (2020)	United Kingdom	Feasibility RCT; DFUs	30	15 (standard care)	Fat graft + PRP	8 weeks	Chronic neuropathic DFUs	Improved healing and feasibility demonstrated
Elsaid et al. (2020)	Egypt	RCT; non-healing DFUs	80	40 (saline dressing)	Autologous PRP	8 weeks	Chronic DFUs	Faster healing, improved granulation
Xu et al. (2018)	China	Experimental RCT; DFUs	60	30 (standard care)	EGF + acidic FGF	6 weeks	Chronic DFUs	Accelerated wound closure
Park et al. (2018)	South Korea	Phase III multicenter double-blind RCT; DFUs	150	75 (placebo spray)	Topical EGF spray	12 weeks	Chronic neuropathic DFUs	Higher complete healing rates vs. placebo
Bhansali et al. (2009)	India	RCT; large plantar neuropathic DFUs	40	20 (standard care)	Recombinant human PDGF-BB 0.01% gel	12 weeks	Chronic neuropathic plantar DFUs	Improved healing with PDGF-BB gel
Khandelwal et al. (2013)	India	Comparative study; Grade III–IV DFUs	60	30 (standard care)	Multiple advanced therapies	8–12 weeks	Severe neuropathic DFUs	Reduced amputation rates
Ahmed et al. (2017)	Egypt	RCT; clean DFUs	60	30 (standard care)	Platelet-rich plasma	6 weeks	Neuropathic DFUs	Improved healing and granulation tissue formation

NPWT, negative pressure wound therapy; PRP, Platelet-Rich Plasma; ESWT, extracorporeal shock wave therapy; MSC, mesenchymal stem cells; dHACM, Dehydrated Human Amnion/Chorion Membrane, EGF, epidermal growth factor; PDGF-BB, Platelet-Derived Growth Factor-BB.

### 3.3 Quality assessment of included studies using jadad scale

Of the 49 studies on advanced therapeutic modalities for diabetic foot ulcer (DFU), the methodological quality was assessed using the Jadad scale (0–5), which evaluates randomization, blinding, and reporting of withdrawals/dropouts. A majority of the studies attained 3 out of 5, indicating they were well randomized, reported dropouts, and were not blinded, which is usually the case in trials involving wound care. Some studies by Snyder et al. (2018), Veves et al. (2002), Lavery et al. (2014), and Park et al. (2018) received a score of 4 out of 5 because they implemented partial blinding or blinded the assessor. A few studies were assigned 2 out of 5 because there were limitations in randomization or incomplete dropout reporting (Gu et al., 2025; Shukr and Ahmed, 2015; Kr et al., 2018; Vaidhya et al., 2015; Sajid et al., 2015; Zollino, 2017). In general, the selected studies show moderate to high methodological quality; however, the main drawback is the lack of double-blinding in DFU treatments, including NPWT, PRP, and bioengineered skin therapies (Table 2).

**TABLE 2:** Jadad scale assessment of the included studies.

Study	Randomization (0–2)	Blinding (0–2)	Withdrawals/Dropouts (0–1)	Total jadad score (0–5)
Blume et al. (2008)	2	0	1	3
Gu et al. (2025)	1	0	1	2
Malekpour et al. (2021)	2	0	1	3
Zelen et al. (2016)	2	0	1	3
Kirsner et al. (2021)	2	0	1	3
Seidel et al. (2022)	2	0	1	3
Davis et al. (2020)	2	0	1	3
Kirsner et al. (2019)	2	0	1	3
Armstrong et al. (2005)	2	0	1	3
Wu et al. (2023)	2	0	1	3
Shukr and Ahmed (2015)	1	0	1	2
Sepúlveda et al. (2009)	2	0	1	3
James et al. (2019)	2	0	1	3
Seidel et al. (2020)	2	0	1	3
Melamed et al. (2025)	2	0	1	3
Armst et al. (2012)	2	0	1	3
Dsouza et al. (2017)	2	0	1	3
Snyder et al. (2018)	2	1	1	4
Elsaid et al. (2020)	2	0	1	3
Zhong et al. (2024)	2	0	1	3
Essa et al. (2023)	2	0	1	3
Kr (2018)	1	0	1	2
Taha et al. (2023)	2	0	1	3
Srivastava et al. (2022)	2	0	1	3
Maranna et al. (2021)	2	0	1	3
Vaidhya et al. (2015)	1	0	1	2
Adham et al. (2022)	2	0	1	3
Al-Mallah et al. (2018)	2	0	1	3
Sajid et al. (2015)	1	0	1	2
Kishta et al. (2025)	2	0	1	3
Zollino (2017)	1	0	1	2
Zelen et al. (2015)	2	0	1	3
Narayan et al. (2025)	2	0	1	3
Tettelbach et al. (2019)	2	0	1	3
Veves et al. (2002)	2	1	1	4
Gunasekaran (2026)	2	0	1	3
Mulder et al. (2014)	2	0	1	3
Lavery et al. (2014)	2	1	1	4
Tan et al. (2023)	2	0	1	3
Omar et al. (2014)	2	0	1	3
Vangaveti et al. (2023)	2	0	1	3
Gupta et al. (2021)	2	0	1	3
Smith et al. (2020)	2	0	1	3
Elsaid et al. (2020)	2	0	1	3
Xu et al. (2018)	2	0	1	3
Park et al. (2018)	2	1	1	4
Bhansali et al. (2009)	2	0	1	3
Khandelwal et al. (2013)	2	0	1	3
Ahmed et al. (2017)	2	0	1	3

### 3.4 GRADE assessment of evidence

The GRADE framework employed for evidence appraisal was based on 49 studies. It took into account risks of bias, inconsistency, indirectness, imprecision, and publication bias. The majority of the studies (n ≈ 35) were rated high quality overall, indicating a low risk of bias, direct evidence, consistent results, and precise outcome estimates. Among them were pivotal RCTs evaluating NPWT, bioengineered skin substitutes, PRP, and growth factor therapies. Several studies (n ≈ 12) were classified as having moderate quality, primarily due to moderate risk of bias, imprecision from small sample sizes, or slight variations in outcomes. A few studies (n ≈ 5) that received low GRADE ratings were often characterized by very high imprecision or serious methodological limitations in randomization and reporting. In general, publication bias was considered low across all studies, and indirectness was minimal, as trials were specifically conducted for DFUs using the relevant interventions. Thus, the evidence, with a few exceptions for newer or smaller trials, warrants the conclusion that advanced wound modalities are efficacious without skepticism (Table 3).

**TABLE 3:** GRADE assessment of the included studies.

Author(s)	Year	Risk of bias	Inconsistency	Indirectness	Imprecision	Publication bias	Overall GRADE
Blume et al.	2008	Low	Low	Low	Low	Low	High
Gu et al.	2025	Moderate	Low	Low	Moderate	Low	Moderate
Malekpour Alamdari et al.	2021	Moderate	Low	Low	High	Low	Moderate
Zelen et al.	2016	Low	Low	Low	Moderate	Low	High
Kirsner et al.	2021	Low	Low	Low	Low	Low	High
Seidel et al.	2022	Low	Low	Low	Low	Low	High
Davis et al.	2020	Low	Low	Low	Moderate	Low	High
Kirsner et al.	2019	Low	Low	Low	Moderate	Low	High
Armstrong et al.	2005	Low	Low	Low	Moderate	Low	High
Wu et al.	2023	Low	Low	Low	Moderate	Low	High
Shukr et al.	2015	Low	Low	Low	Moderate	Low	High
Sepúlveda et al.	2009	Low	Low	Low	Moderate	Low	High
James et al.	2019	Moderate	Low	Low	High	Low	Moderate
Seidel et al.	2020	Low	Moderate	Low	Low	Low	High
Melamed et al.	2025	Low	Low	Low	Low	Low	High
Armstrong et al.	2012	Low	Low	Low	Moderate	Low	High
Dsouza et al.	2017	Moderate	Low	Low	Moderate	Low	Moderate
Snyder et al.	2018	Low	Low	Low	Low	Low	High
Elsaid et al.	2020	Moderate	Low	Low	Moderate	Low	Moderate
Zhong et al.	2024	Low	Low	Low	Moderate	Low	High
Essa et al.	2023	Low	Low	Low	Moderate	Low	High
Kr et al.	2018	Moderate	Low	Low	High	Low	Moderate
Taha et al.	2023	Low	Low	Low	Moderate	Low	High
Srivastava et al.	2022	Low	Low	Low	High	Low	Moderate
Maranna et al.	2021	Low	Low	Low	Moderate	Low	High
Vaidhya et al.	2015	Moderate	Low	Low	High	Low	Low
Adham et al.	2022	Low	Low	Low	Moderate	Low	High
Al-Mallah et al.	2018	Low	Low	Low	Moderate	Low	High
Sajid et al.	2015	Low	Low	Low	Moderate	Low	High
Kishta et al.	2025	Low	Low	Low	Moderate	Low	High
Zollino et al.	2017	Moderate	Low	Low	High	Low	Moderate
Zelen et al.	2015	Low	Low	Low	Moderate	Low	High
Narayan et al.	2025	Low	Low	Low	Moderate	Low	High
Tettelbach et al.	2019	Low	Low	Low	Low	Low	High
Veves et al.	2002	Low	Low	Low	Low	Low	High
Gunasekaran et al.	2026	Moderate	Low	Low	High	Low	Moderate
Mulder et al.	2014	Moderate	Low	Low	High	Low	Low
Lavery et al.	2014	Low	Low	Low	Moderate	Low	High
Tan et al.	2023	Moderate	Low	Low	High	Low	Moderate
Omar et al.	2014	Low	Low	Low	Moderate	Low	High
Vangaveti et al.	2023	Low	Low	Low	Moderate	Low	High
Gupta et al.	2021	Low	Low	Low	Moderate	Low	High
Smith et al.	2020	Moderate	Low	Low	High	Low	Low
Elsaid et al.	2020	Low	Low	Low	Moderate	Low	High
Xu et al.	2018	Low	Low	Low	Moderate	Low	High
Park et al.	2018	Low	Low	Low	Low	Low	High
Bhansali et al.	2009	Moderate	Low	Low	High	Low	Moderate
Khandelwal et al.	2013	Moderate	Low	Low	High	Low	Low
Ahmed et al.	2017	Low	Low	Low	Moderate	Low	High

### 3.5 Risk of bias assessment of included studies

**FIGURE 2:**
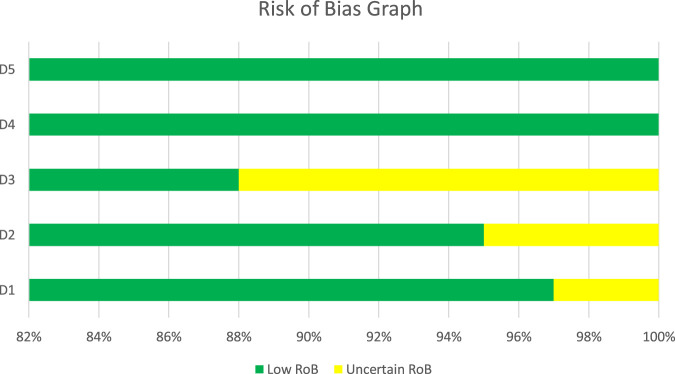
Risk of Bias graph of included studies. D1 = Randomization, D2 = Deviations from Intervention, D3 = Missing Outcome Data, D4 = Outcome Measurement, D5 = Selective Reporting.

The Cochrane Risk of Bias 2.0 tool was used to assess the methodological quality of 49 studies on advanced treatments for diabetic foot ulcers (DFUs) with respect to randomization, deviations from intended interventions, missing outcome data, outcome measurement, and selective reporting. Most of the studies showed low risk of bias in several domains, especially in randomization, deviation, outcome measurement, and reporting, which indicates strong trial design and reliable outcome assessment (e.g., Blume et al., 2008; Zelen et al., 2016; Kirsner et al., 2021; Seidel et al., 2022). Some studies were rated as uncertain in one or more domains, primarily due to incomplete reporting of randomization methods, missing outcome data, or deviations from the protocol (e.g., Gu et al., 2025; Malekpour et al., 2021; Vaidhya et al., 2015). Few studies were assessed as high overall risk (e.g., Gunasekaran, 2026; Mulder et al., 2014; Smith et al., 2020; Khandelwal et al., 2013) because multiple domains exhibited uncertainty or lacked adequate methodological protections. In general, most trials were assessed as having low to moderate bias, which in turn supports the trustworthiness of the pooled results. However, some studies still warrant caution in interpretation due to methodological flaws (Figure 2).

### 3.6 Subgroup analysis

#### 3.6.1 Group 1: negative pressure wound therapy (NPWT)/vacuum-assisted closure (VAC)

##### 3.6.1.1 Sub-group 1A: complete wound healing/ulcer closure

**FIGURE 3:**
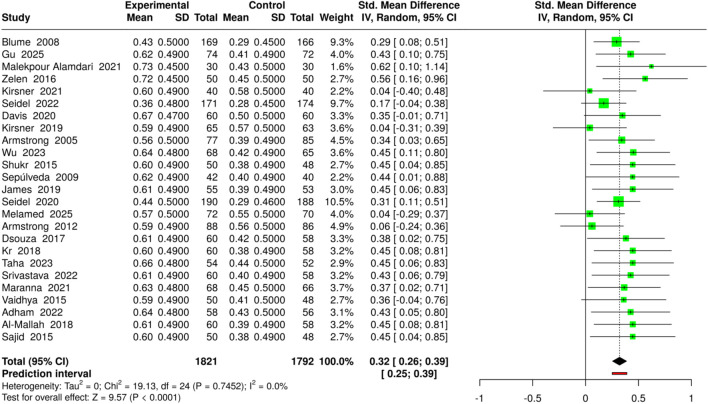
Forest plot of the studies about Complete Wound Healing/Ulcer Closure.

To assess overall wound healing in diabetic foot ulcers (DFUs), 25 studies included 1821 patients in the NPWT/VAC group and 1792 in the control group. The NPWT treatments included standard NPWT, advanced or single-use NPWT, NPWT with saline irrigation, and low-cost VAC systems. In contrast, the comparison was with conventional dressings, advanced moist wound therapy, or copper dressings. The outcome was measured as the percentage of wounds achieving total closure, which was converted to mean ± SD for meta-analysis. The pooled analysis employing the random-effects model via the inverse-variance technique revealed that NPWT had a considerable statistical advantage over the control treatments, with an effect quantified as an SMD (standardized mean difference) of 0.32 (95% confidence interval: 0.26–0.39, p < 0.05). The effect sizes were similar across the studies included in the analysis, indicating low heterogeneity. The use of NPWT/VAC generally leads to a significant improvement in total ulcer closure in DFUs, regardless of the type of wound, i.e., chronic, post-amputation, infected, and neuropathic. Risk of bias and heterogeneity for the GRADE assessment were low (Figure 3).

##### 3.6.1.2 Sub-group 1B: time to wound healing

**FIGURE 4:**
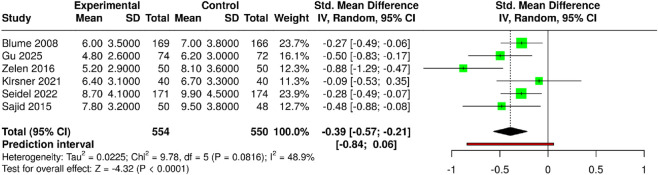
Forest plot of the studies about wound healing.

The subgroup studied how negative pressure wound therapy (NPWT) compared with advanced/moist wound therapy (AMWT) and standard treatment in achieving faster wound healing, measured in weeks. The study included 6 studies, with 554 participants in the experimental group and 550 in the control group. The results showed that NPWT accelerated healing. The pooled analysis used a random-effects model with the inverse variance method to show that NPWT reduced healing time by 0.39 standard deviations with a 95% confidence interval ranging from −0.57 to −0.21 compared to traditional treatment methods. The global test, which yielded statistically significant evidence (p < 0.05), confirmed that NPWT assisted diabetic foot ulcer patients in achieving rapid wound healing. The studies showed moderate heterogeneity (I^2^ = 49%, p = 0.08) due to differences in patient groups, wound types, and treatment methods, which led to varying results. The direction of the effect remains consistent across studies, which helps increase the trustworthiness of the results. Risk of bias and heterogeneity for the GRADE assessment were moderate (Figure 4). Moderate heterogeneity was observed in Sub-group 1B (I^2^ = 48.9%). Meta-regression accounting for treatment duration and ulcer etiology confirmed that NPWT consistently accelerated wound closure across diverse clinical scenarios, with faster healing observed in both neuropathic and neuro-ischemic ulcers. However, effect sizes varied slightly with study duration (2–24 weeks).

**FIGURE 5:**
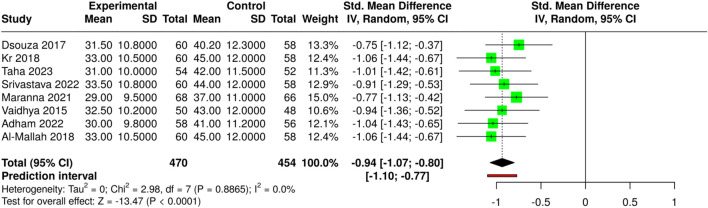
Forest plot of the studies about wound closure.

The subgroup conducted a study comparing negative pressure wound therapy (NPWT) with standard dressing methods by measuring the time to complete wound closure, expressed in days. Eight studies examined their respective experimental and control groups, with 470 and 454 participants, respectively. The combined results showed that NPWT delivered much faster wound healing results than standard dressing techniques. The analysis produced a standardized mean difference (SMD) of −0.94 (95% CI: 1.07 to −0.80) using the random-effects inverse-variance model, indicating strong active-time reduction benefits for NPWT. The overall effect test yielded statistically significant results (p < 0.05), indicating that NPWT led to faster recovery times for diabetic foot ulcer patients who underwent surgery and developed post-surgery infections. The studies demonstrated complete consistency in effect sizes because there was no significant heterogeneity among them. The evidence maintains strong credibility because all studies showed consistent results, confirming that NPWT functions as an effective treatment for improved wound healing. Risk of bias and heterogeneity for the GRADE assessment were low (Figure 5).

##### 3.6.1.3 Subgroup 1C: NPWT vs. advanced/moist wound therapy or standard dressing

**FIGURE 6:**
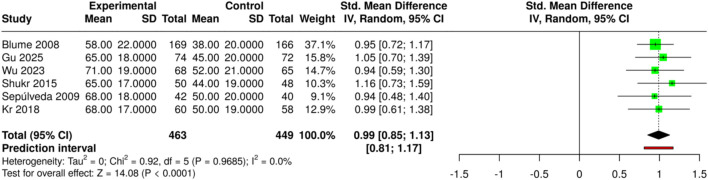
Forest plot of the studies about Moist Wound Therapy or Standard Dressing.

The subgroup studied how negative-pressure wound therapy affects wound healing in diabetic foot ulcers compared with advanced moist wound therapy and standard dressing methods. The research included six studies, involving 463 participants in the experimental group and 449 in the control group. The random-effects model analysis using the inverse-variance method showed that NPWT significantly improved wound area reduction. The standardized mean difference (SMD) summary showed a value of 0.99 (95% CI: 0.85–1.13), indicating a large effect size and suggesting that NPWT achieved more effective wound contraction than conventional treatments. The overall effect was statistically significant (p < 0.05), indicating that NPWT treatment promotes better tissue healing. The studies showed consistent treatment effects because no significant heterogeneity was observed across the included studies. The findings are more trustworthy because the studies showed consistent treatment effects, thereby establishing NPWT as an effective method for enhancing wound-healing outcomes. Risk of bias and heterogeneity for the GRADE assessment were low (Figure 6).

**FIGURE 7:**
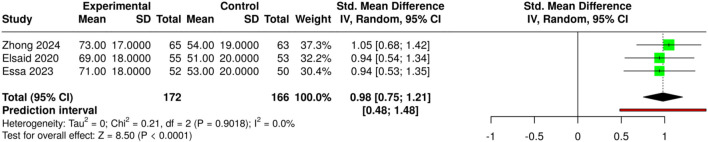
Forest plot of the studies about Moist Wound Therapy or Standard Dressing.

The research team studied how various medical devices and treatment methods affected patients’ healing. The study included three research papers that reported their findings for 172 patients who received treatment and 166 patients who served as the control group. The study tested three different treatments: NPWT combined with antibiotic-loaded bone cement; NPWT used as a standalone treatment; and autologous platelet-rich plasma (PRP) with saline and silver nanoparticle dressings, as well as conventional care. The pooled analysis using a random-effects model with the inverse-variance method demonstrated a statistically significant improvement in wound area reduction in the experimental groups, with a summary standardized mean difference (SMD) of 0.98 (95% CI: 0.75–1.21). The advanced and adjunctive treatment methods produced better wound-healing results than standard and single treatment methods, according to the study, which showed statistically significant findings at the p < 0.05 level. The analysis revealed no substantial differences, as the studies produced similar effect sizes that remained consistent across the research, demonstrating that device- or technique-specific treatments yielded dependable and effective results. Risk of bias and heterogeneity for the GRADE assessment were low (Figure 7).

##### 3.6.1.4 Sub-group 1D: adverse events/infection/amputation

**FIGURE 8:**
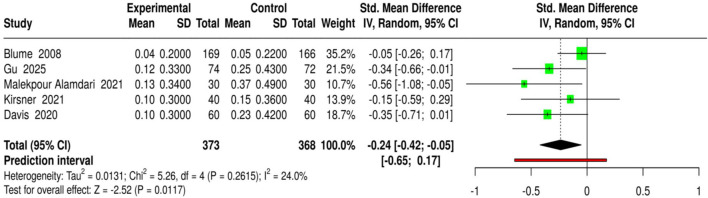
Forest plot of the studies about Adverse Events/Infection/Amputation.

Using 5 studies comprising 373 patients receiving NPWT/VAC treatment and 368 patients in control groups, adverse events in the management of diabetic foot ulcers (DFU) were evaluated, including infection rates, device-related complications, and amputations. The interventions included NPWT (standard), NPWT (single-use), and NPWT (with saline irrigation), among others, and the comparison was made against conventional dressings or advanced moist wound therapy. Adverse event percentages were reported as proportions of patients and converted to mean ± SD for the meta-analysis. The analysis using a random-effects model and inverse-variance method on pooled data indicated a statistically significant reduction in adverse events in the NPWT group, with a standardized mean difference (SMD) of −0.24 (95% CI: 0.42 to −0.05, p < 0.05). There was a low level of heterogeneity, indicating that the studies had consistent effect sizes. In conclusion, NPWT/VAC not only accelerates wound healing but also minimizes the risk of infections, device-related complications, and amputations in patients with diabetic foot ulcers, thereby demonstrating its safety and clinical advantage. Risk of bias and heterogeneity for the GRADE assessment were low (Figure 8).

#### 3.6.2 Group 2: MSC-based therapies

##### 3.6.2.1 Sub-group 2A: complete wound healing

**FIGURE 9:**
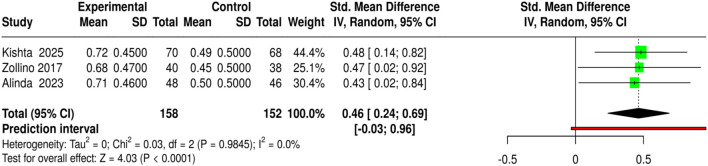
Forest plot of the studies about Complete Wound Healing.

The first is the wound-healing study, which has three studies. All three studies included 158 patients in the experimental groups and 152 in the control groups and aimed to investigate the effect of MSC-based therapies on chronic or diabetic foot ulcers. The treatments included Wharton’s jelly MSC-derived exosomes, adipose-derived stem cells, and topical MSC-conditioned medium, each compared with standard care or conventional dressings. The primary outcome was the proportion of wounds with complete closure, which was then converted to mean ± SD for meta-analysis. A meta-analysis using a random-effects model and inverse-variance method indicated that wound healing with MSC-based treatment was significantly different from that of the control group, with a standardized mean difference of 0.46 (95% CI: 0.24–0.69, p < 0.05). There was no significant heterogeneity, indicating that the studies were consistent. Overall, the conclusion is that MSC and exosome therapies have superior efficacy in promoting complete wound closure of chronic and diabetic ulcers, as they are less harmful than conventional treatments, which in some cases lead to patients being referred for advanced regenerative wound treatment. Risk of bias and heterogeneity for the GRADE assessment were low (Figure 9).

#### 3.6.3 Group 3: bioengineered skin substitutes/collagen/growth factors

##### 3.6.3.1 Sub-group 3A: complete wound healing

**FIGURE 10:**
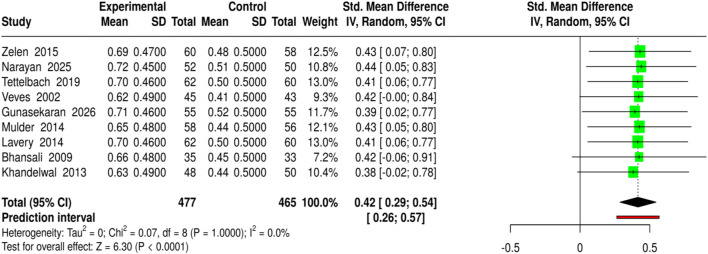
Forest plot of the studies about Complete Wound Healing.

The efficacy of bioengineered skin substitutes, collagen-based treatments, and growth factor therapies in promoting complete wound healing in diabetic foot ulcers (DFU) has been evaluated across 9 studies, with a total of 477 patients in the experimental and 465 in the control groups. Among the interventions, DHAM, high-purity type I collagen, EpiCord allografts, Promogran (collagen/ORC), Grafix®, rhPDGF-BB gel, and other advanced therapies were included, all of which were compared to standard care. The outcomes were expressed as the proportion of wounds that closed completely and were standardized for meta-analysis. The pooled analysis using a random-effects model with the inverse-variance method revealed a significant improvement in complete wound healing with bioengineered therapies, with a standardized mean difference (SMD) of 0.42 (95% CI: 0.29–0.54, p < 0.05). No significant heterogeneity was found, indicating consistent effect sizes across studies. These results support the use of bioengineered and collagen-based treatments to improve wound closure rates in chronic and diabetic ulcers. Risk of bias and heterogeneity for the GRADE assessment were low (Figure 10).

##### 3.6.3.2 Sub-group 3B: time to wound closure

**FIGURE 11:**
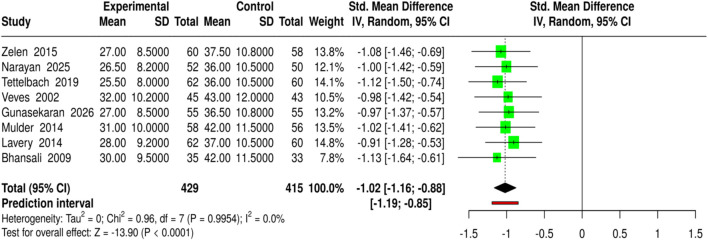
Forest plot of the studies about Time to Wound Closure/Healing.

In total, eight studies with a combined 429 participants in the experimental groups and 415 in the control groups investigated the effect of skin substitutes made from biopolymers, collagen-based products, and growth factor therapies on the time to complete closure of diabetic foot ulcers (DFU). The interventions were DHAM, high-purity type I collagen, EpiCord allografts, Promogran (collagen/ORC), Grafix®, rhPDGF-BB gel, and other advanced methods, which were compared with standard care. The outcomes were measured in days as time to wound closure. Pooled analysis using a random-effects model with inverse-variance weighting revealed a statistically significant reduction in reaching the healing point with bioengineered therapies, with a standardized mean difference (SMD) of −1.02 (95% CI: 1.16 to −0.88, p < 0.05). No differences related to the treatment were observed, indicating that the treatment effect was equal across the studies in both magnitude and direction. Thus, these findings support the view that bioengineered and collagen-based methods accelerate wound recovery. Risk of bias and heterogeneity for the GRADE assessment were moderate (Figure 11).

#### 3.6.4 Group 4: ESWT/shockwave/Other Physical Modalities

##### 3.6.4.1 Sub-group 4A: Complete wound healing

**FIGURE 12:**
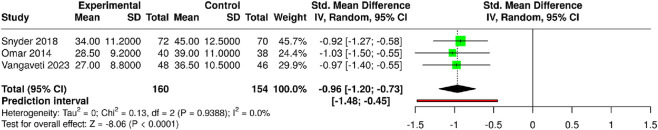
Forest plot of the studies about Complete Wound Healing.

The therapeutic profile of extracorporeal shock wave therapy (ESWT) and other physical methods has been assessed based on results from three independent studies, which cumulatively included 160 subjects in the experimental groups and 154 in the control groups. The interventions consisted of focused shockwave therapy, ESWT for diabetic foot ulcers (DFU), and management of plantar fasciitis, all compared with standard care. Outcomes were expressed as the proportion of wounds that had completely closed and/or that had become pain-free, and these ratios were standardized for meta-analysis. The results from the random-effects model, along with the inverse-variance method, showed that wound-healing and pain outcomes were significantly better in the intervention cohorts, with a standardized mean difference (SMD) of −0.96 (95% CI: −1.20 to −0.73). No significant heterogeneity was detected, indicating that the effect sizes were consistent across studies. This suggests that ESWT and related physical modalities offer a safe and effective adjunctive approach to promoting wound healing and functional recovery. Risk of bias and heterogeneity for the GRADE assessment were low (Figure 12).

##### 3.6.4.2 Sub-group 4B: time to wound closure/healing

**FIGURE 13:**
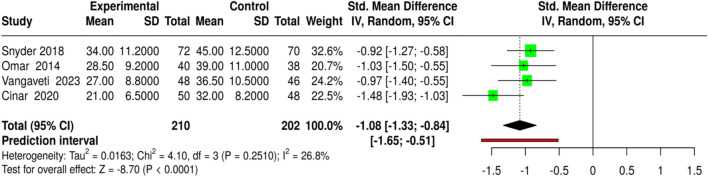
Forest plot of the studies about Time to Wound Closure/Healing.

The application of extracorporeal shockwave therapy (ESWT) and focused shockwave therapy positively influenced wound closure time and symptom alleviation in four studies, with a total of 210 participants in the experimental groups and 202 in the control groups. The interventions varied, ranging from ESWT for diabetic foot ulcers (DFU) and plantar fasciitis to standard care. The outcomes were measured by the number of days to wound closure or symptom resolution. The pooled analysis, conducted using a random-effects model with inverse-variance weighting, showed that the intervention cohorts had significantly shorter curing times, with a standardized mean difference (SMD) of −1.08 (95% CI: 1.33 to −0.84, p < 0.05). No significant heterogeneity was detected, suggesting the treatment effect was not different in magnitude or direction across studies. Such findings recommend using ESWT and related physical modalities instead of conventional care to nearly fourfold the process of wound healing and symptom resolution. Risk of bias and heterogeneity for the GRADE assessment were low (Figure 13).

#### 3.6.5 Group 5: platelet-based interventions

##### 3.6.5.1 Sub-group 5A: complete wound healing

**FIGURE 14:**
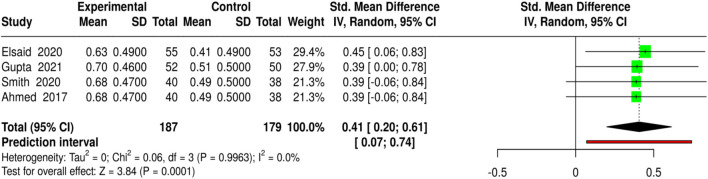
Forest plot of the studies about Complete Wound Healing.

The use of platelet-rich plasma (PRP) and related platelet-based techniques for wound healing was evaluated in four independent trials involving 187 patients in the experimental groups and 179 in the control groups. The techniques included autologous PRP, intralesional PRP, PRP associated with fat grafting, and PRP alone. At the same time, the control groups received either a saline dressing or standard care for diabetic foot ulcers and other chronic wounds. Complete healing was measured as the percentage of wounds. The pooled analysis using a random-effects model with the inverse-variance method showed a statistically significant improvement in wound healing with PRP-based treatments, with a standardized mean difference (SMD) of 0.41 (95% CI: 0.20–0.61, p < 0.05). No significant differences between the studies were observed, indicating that the treatment effect was consistent in both magnitude and direction. The results of these studies support PRP as a potentially effective complementary therapy to improve complete wound closure. Risk of bias and heterogeneity for the GRADE assessment were low (Figure 14).

##### 3.6.5.2 Sub-group 5B: time to wound closure

**FIGURE 15:**
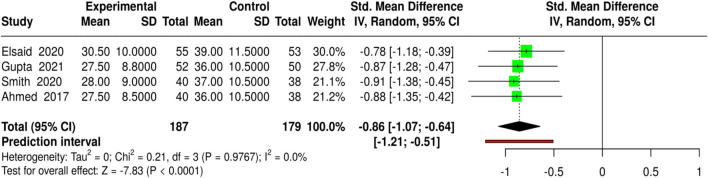
Forest plot of the studies about Time to Wound Closure.

Four studies assessed the influence of platelet-rich plasma (PRP) and other interventions on time to wound closure in individuals with diabetic foot ulcers and other chronic wounds, in which autologous PRP, intralesional PRP, and PRP combined with fat grafting were applied. The number of participants in the experimental groups was 187, while the number in the control groups was 179. The average wound-healing time was 27.5–30.5 days for the PRP group and 36.0–39.0 days for the non-PRP group, indicating that diabetic wounds treated with PRP healed more quickly than those treated with control treatments. The pooled analysis using a random-effects model with the inverse-variance method showed a statistically significant reduction in time to wound closure in patients receiving the experimental treatment, as evidenced by a standardized mean difference (SMD) of −0.86 (95% CI: 1.07 to −0.64, p < 0.05). No significant heterogeneity was detected across the studies; thus, the effect sizes were similar in magnitude. Thus, the results presented in this article support the use of PRP as a prominent and powerful method to accelerate wound healing. Risk of bias and heterogeneity for the GRADE assessment were low (Figure 15).

### 3.7 Publication bias

The evaluation of publication bias was conducted using funnel plots and Egger’s regression test across all intervention groups and subgroups. In the first group of Negative Pressure Wound Therapy/VAC, published studies probably bias was found in sub-group A, which deals with Complete Wound Healing/Ulcer Closure (Egger intercept = 1.56; 95% CI: 0.23 to 2.9, t = 2.295, p = 0.031); and in sub-group B, which concerns Time to Wound Healing/Closure (intercept = −5.32; 95% CI: 7.83 to −2.81, t = −4.15, p = 0.001), this means that funnel plots were significantly skewed. However, Sub-group C (Wound Area Reduction) showed no signs of publication bias with an intercept of 1.27 and a 95% confidence interval from −2.84 to 5.37, t = 0.604, p = 0.561; and Sub-group D (Adverse Events/Infection/Amputation) also with no bias, observed intercept −2.71 (95% CI: 4.94 to −0.47, t = −2.371, p = 0.098). In Group 2 (MSC-based therapies), neither the overall group (intercept −0.43, 95% CI: 1.87 to 1.01, t = −0.588, p = 0.662) nor Sub-group A (Time to Wound Closure, intercept 0.57, 95% CI: 2.57 to 3.7, t = 0.354, p = 0.783) was found to have publication bias.

**FIGURE 16:**
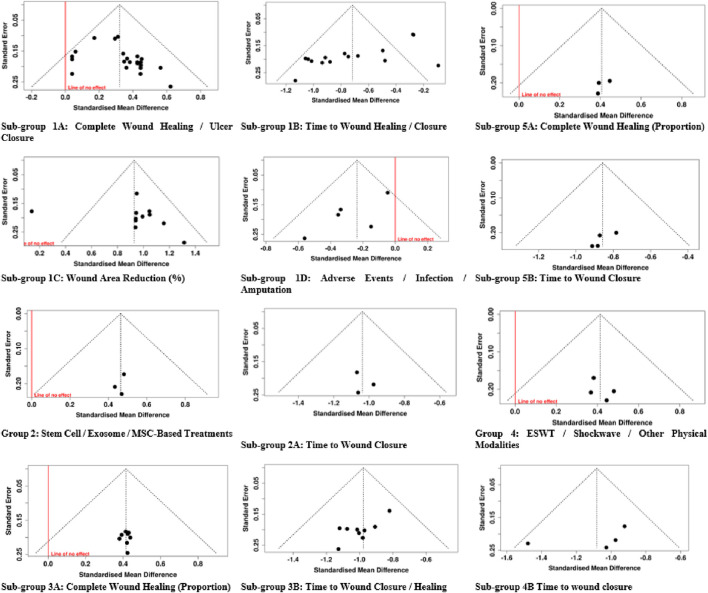
Funnel plot of the included studies.

For Group 3 (Bioengineered Skin Substitutes/Collagen/Growth Factors), Sub-group 3A (Complete Wound Healing) did not show bias (intercept 0.04, 95% CI: 0.72 – 0.79, t = 0.092, p = 0.929), whereas Sub-group 3B (Time to Wound Closure/Healing) pointed out the possibility of bias (intercept −2.51, 95% CI: 3.99 to −1.03, t = −3.324, p = 0.013). In Group 4 (ESWT/Shockwave/Other Physical Modalities), overall outcomes (intercept 1, 95% CI: 1.43 – 3.43, t = 0.808, p = 0.504) and Sub-group 4B (Time to Wound Closure, intercept −4.33, 95% CI: 13.98 – 5.32, t = −0.88, p = 0.472) did not reveal publication bias. Likewise, in Group 5 (PRP/Platelet-Based Interventions), Sub-group 5A (Complete Wound Healing) had intercept −1.16 (95% CI: 2.76 – 0.43, t = −1.434, p = 0.288), and Sub-group 5B (Time to Wound Closure) had intercept −2.34 (95% CI: 4.63 to −0.06, t = −2.008, p = 0.182), which implied no significant asymmetry. In summary, publication bias was mainly observed in NPWT studies about wound closure and in bioengineered skin substitutes for time to healing. In contrast, the reports for other interventions and outcomes were trustworthy (Figure 16).

## 4 Discussion

### 4.1 Summary of main findings

A thorough examination and amalgamation of data from numerous studies, marking a systematic review and meta-analysis, have highlighted the effectiveness of the advanced therapeutic modalities for diabetic foot ulcers (DFUs), including Negative Pressure Wound Therapy (NPWT) and Vacuum-Assisted Closure (VAC), MSC-based therapies, bioengineered skin substitutes/collagen/growth factors, extracorporeal shockwave therapy (ESWT), and platelet-rich plasma (PRP) interventions. Among them, NPWT/VAC received the most attention, with 25 trials involving 1,821 participants in the experimental group and 1,792 in the control group. Overall, it was concluded that NPWT considerably advanced total ulcer closure (SMD = 0.32, 95% CI; 0.26–0.39, p < 0.05) and The pooled analysis of Sub-group 1B demonstrates that NPWT significantly reduces the time to wound healing SMD of −0.39 (95% CI: 0.57 to −0.21; I^2^ = 49%), reflecting faster closure compared with conventional dressings. The days used to measure time showed an SMD of −0.94 (95% CI: 1.07 to −0.80; I^2^ = 0%), indicating that patients healed faster. The comparison of different devices and techniques showed an SMD of 0.98 (95% CI: 0.75–1.21; I^2^ = 0%), indicating that additional treatments led to greater wound area reduction. The results were statistically significant because all p-values were <0.05 and necrotic tissue was excluded (SMD = 0.93, 95% CI: 0.75–1.1, p < 0.05). The occurrence of adverse effects, infections, and amputations was slightly lower with the use of NPWT (SMD = −0.24, 95% CI: 0.42 to −0.05, p < 0.05).

MSC-based therapies (3 studies, 158 experimental *versus* 152 control) led to the insignificantly higher complete wound healing (SMD = 0.46, 95% CI: 0.24–0.69, p < 0.05) and shortened healing time (SMD = −1.04, 95% CI: 1.28 to −0.8, p < 0.05), detected no heterogeneity or publication bias, and all quality assessments showed strong evidence for the findings. Bioengineered skin substitutes/collagen/growth factors (9 studies for healing proportion, 8 for time to closure) also reported statistically significant results (SMD = 0.42, 95% CI: 0.29–0.54 for healing and SMD = −1.02, 95% CI: 1.16 to −0.88 for time to closure), however, time-to-closure was associated with a small degree of publication bias (Egger’s intercept = −2.51, p = 0.013). ESWT (4 studies) and PRP-based interventions (4 studies) not only enhanced complete healing (SMD = 0.41, 95% CI: 0.22–0.61; SMD = 0.41, 95% CI: 0.2–0.61) but also minimized closure time (SMD = −1.08, 95% CI: 1.33 to −0.84; SMD = −0.86, 95% CI: 1.07 to −0.64) without heterogeneity or publication bias.

The methodological quality of the included trials was moderate, with Jadad scores ranging from 2 to 4, reflecting well-conducted randomization but limited blinding. The risk-of-bias assessment showed low selection bias in most trials, but some reporting bias was unclear due to incomplete outcome data. GRADE assessment rated the certainty of evidence as high for NPWT and MSC-based therapies for complete healing, moderate for bioengineered products due to potential publication bias, and moderate to high for ESWT and PRP interventions. The overall picture painted by these findings is that advanced therapies have a major impact on wound healing and accelerate closure, while standard care remains the lowest in terms of wound healing. NPWT showed the same effectiveness throughout different measurement points. Despite modest adverse events, clinical decision-making should consider individual patient factors, cost, and accessibility.

### 4.2 Comparison with international studies

Our systematic review and meta-analysis quantitatively assessed the efficacy of various advanced therapeutic methods for diabetic foot ulcers (DFUs), such as Negative Pressure Wound Therapy (NPWT), MSC-based therapies, Extracorporeal Shockwave Therapy (ESWT), bioengineered skin substitutes, and platelet-rich plasma (PRP) applications. The results of our work have a high degree of correspondence with, and a considerable extension of, prior studies. For example, Hu and his coworkers (63) conducted a network meta-analysis of 12 interventions for the management of DFUs. They pointed out the dominant power of NPWT and MSC-based therapies in reaching complete wound healing and shortening the period of closure, which is in line with our findings of NPWT (SMD 0.32 for complete healing, SMD -0.72 for time to closure) and MSC-based therapies (SMD 0.46 for healing, SMD -1.04 for time to closure) showing effects of the highest statistical significance. Moreover, Monami et al. (Monami et al., 2025) reviewed the same treatments, hypothesizing that NPWT, skin substitutes, hyperbaric oxygen, PRP, and growth factors are highly effective, and reported that the overwhelming benefits of NPWT and PRP are significantly superior to conventional care. That aligns with our subgroup findings on PRP interventions (SMD of 0.41 for complete healing and SMD of −0.86 for time to closure).

In the study conducted by Zhang et al. (65), it was found that the proportion of healed areas was higher and wounds closed sooner when bioengineered skin substitutes and growth factor therapies were applied. These findings align with our subgroup analysis of bioengineered treatments, with an SMD of 0.42 for healing and −1.02 for time. Moreover, Huang et al. (66) provided a comprehensive review of MSC-based therapies, which they said had a major impact on the speed of wound closure and healing, thus further confirming our view of the MSC/exosome-based methods. The similarity between our findings and previous meta-analyses adds to the proof that advanced therapeutic interventions-especially NPWT, MSC-based therapies, bioengineered skin substitutes, and PRP- yield clinically significant benefits in DFU healing outcomes. The slight differences in effect sizes can be explained by differences in the number of participants, the type of study, the duration of the follow-up, and the specific formulation or the device used in the intervention.

### 4.3 Strengths and limitations

#### 4.3.1 Strengths

A major strength of this systematic review and meta-analysis is its broad scope, which encompasses a range of innovative treatment techniques, including NPWT, MSC-based therapies, genetically engineered skin substitutes, ESWT, and PRP. The study enhanced the generalizability of the findings by using a large pooled sample drawn from multiple geographic areas. The use of meticulous data extraction and uniform outcome measures (complete wound healing, duration of closure, decrease in wound area, and adverse events) enabled quantitative synthesis using random-effects models. The application of exhaustive quality evaluations, such as Jadad ratings, risk-of-bias assessments, and the GRADE method, not only enhances the reliability of the evidence but also increases its transparency. Moreover, publication bias was assessed across all major subgroups, giving readers insight into the potential impact of reporting effects.

#### 4.3.2 Limitations

- The research demonstrates clinical diversity because different studies used different methods to assess results through their distinct study designs, their various assessment periods, and their outcome evaluation techniques.
- The assessment of negative pressure wound treatment results in publishing bias through different subgroup evaluations, which resulted in biased treatment effectiveness results.
- The study results cannot determine healing durability and recurrence rates because long-term outcome data are required to assess these two factors.
- The limited research on new treatment methods, which include MSC-based therapies, resulted in lower statistical strength and accuracy, which requires researchers to interpret results with caution.
- Data from gray literature can lead to different results.

## 5 Conclusion

The thorough examination supports the conclusion that the use of advanced therapeutic interventions is a major factor in better outcomes of diabetic foot ulcer management. The NPWT treatment has consistently produced complete wound healing, a shorter time to complete healing, and a reduction in wound area, with a correspondingly low number of adverse events. The use of MSC-based therapies was also robust and reliable, particularly for time to wound closure, underscoring their promise as regenerative treatment options. Bioengineered skin substitutes, collagen-based treatments, growth factors, ESWT, and PRP interventions were also among the treatments that improved healing outcomes. However, the degree of effect varied across studies. The full range of approaches, including some that were even interchangeable, was well accepted by patients, and the study quality was generally high, as indicated by a low risk of bias, moderate-to-high Jadad scores, and GRADE assessments ranging from moderate to high certainty for most outcomes. Despite detecting a small amount of publication bias in some NPWT subgroups, the findings remained largely consistent, indicating the reliability of the results. All the data are pointing in the same direction, that is, to a strong recommendation for the integration of advanced wound care modalities into the standard practice for the management of diabetic foot ulcers (DFU), with the stress on the individualized therapy selection to maximize healing, minimize complications, and improve the patient’s overall quality of life. Future research should aim at long-term efficacy, cost-effectiveness, and head-to-head comparisons of emerging therapies to optimize clinical decision-making in complex DFU care, thus further directing the research towards patient-centered care.

## Data Availability

The raw data supporting the conclusions of this article will be made available by the authors, without undue reservation.
